# Adaptor Protein 2 (AP-2) complex is essential for functional axogenesis in hippocampal neurons

**DOI:** 10.1038/srep41620

**Published:** 2017-01-31

**Authors:** Jae Won Kyung, In Ha Cho, Sukmook Lee, Woo Keun Song, Timothy A. Ryan, Michael B. Hoppa, Sung Hyun Kim

**Affiliations:** 1 Department of Biomedical Science, Graduate School, Kyung Hee University, Seoul, 02447, South Korea; 2 Department of Biology, Molecular Cellular Biology Program, Dartmouth College, Hanover, NH, 03755, USA; 3 Laboratory of Molecular Cancer Therapeutics, Scripps Korea Antibody Institute, Chuncheon, 24341, South Korea; 4 School of Life Science, Bioimaging Research Center, Gwangju Institute of Science and Technology (GIST), Gwangju, 61005, South Korea; 5 Department of Biochemistry, Weill Cornell Medical College, New York, NY, 10065, USA; 6 Department of Physiology, Neurodegeneration Control Research Center, School of Medicine, Kyung Hee University, Seoul, 02447, South Korea

## Abstract

The complexity and diversity of a neural network requires regulated elongation and branching of axons, as well as the formation of synapses between neurons. In the present study we explore the role of AP-2, a key endocytic adaptor protein complex, in the development of rat hippocampal neurons. We found that the loss of AP-2 during the early stage of development resulted in impaired axon extension and failed maturation of the axon initial segment (AIS). Normally the AIS performs two tasks in concert, stabilizing neural polarity and generating action potentials. In AP-2 silenced axons polarity is established, however there is a failure to establish action potential firing. Consequently, this impairs activity-driven Ca^2+^ influx and exocytosis at nerve terminals. In contrast, removal of AP-2 from older neurons does not impair axonal growth or signaling and synaptic function. Our data reveal that AP-2 has important roles in functional axogenesis by proper extension of axon as well as the formation of AIS during the early step of neurodevelopment.

In order to properly integrate into functional circuits, neurons must establish cellular polarity including the elaboration of dendritic branches and elongation of a single axon that navigates through guidance cues to establish synaptic terminals. Some of the critical molecular signaling pathways have been identified in axogenesis^1^, path finding^2^, and synaptogenesis^3^. However, less is known about how these systems rely on basic endocytic machinery to function. One would expect endocytosis to be important for a number of stages in neuronal development as precise sorting of cargoes or membrane proteins is essential during development of hippocampal neurons^4^. Forward extension of growth cones in neurites involves continuous addition and retrieval of membrane to drive the leading edge. Furthermore, endocytosis of signaling receptors such as netrin-DCC^5^,^6^, Slit-Robo^7^,^8^, semaphorin-neuropilin^9^, and ephrin-eph receptor^10^ is critical for correct axon guidance and outgrowth^11^,^12^,^13^. Molecules such as AP-2 and clathrin are usually involved in clearing such receptors from the cell surface, similar to the role they play in the delivery of iron through endocytosis of the transferrin receptor.

Another critical aspect of axogenesis, aside from signaling and guidance is establishing subcellular signaling domains. These domains are created by the precise sorting of axonal and dendritic proteins including voltage-gated ion channels^14^,^15^,^16^,^17^. A key stage of this process is the formation of the axon initial segment (AIS). The AIS serves as a barrier to maintain polarity as well as a functional role in signaling by initiating action potentials. The AIS is composed of an array of structural and cytoskeletal proteins which localize to the proximal region of axon helping to establish a barrier for selective transport of cargo to the axon^18^. The signaling features of the AIS are enabled because this region of axon is studded with a precise array of voltage-gated ion channels including a high concentration of voltage-gated sodium (Na_V_) channels to initiate action potentials. At present, the molecular mechanisms enabling the delivery of these ion channels are unknown^19^,^20^. One possibility in neurons is that the enrichment of ion channels along the axon and at the AIS are selectively retrieved from other areas of the cell (proximal dendrites and soma) for subsequent delivery early in polarization similar to signaling receptors. Clathrin-mediated endocytosis has been identified as an important mechanism for enriching Na^+^ channels in epithelial tissue (a classic polarized cell)^21^,^22^. Endocytosis was also identified as a critical process for modulating ligand-gated channels at the postsynaptic density of dendritic spines^23^. Taken together, these observations warrant a closer study of endocytic proteins in neuronal axogenesis and signaling.

Previously, using shRNA-mediated ablation of the μ2-subunit to deplete overall AP-2 complex levels in mature polarized hippocampal neurons, we demonstrated that this clathrin-associated adaptor protein complex is critical for efficient synaptic vesicle endocytosis^24^,^25^. Here we show that removal of AP-2 at early stages of neuronal development impedes axogenesis, prevents accumulation of Na_V_ channels at the AIS, and impairs synaptic transmission. In contrast, removal of AP-2 once the axon has formed does not alter signal propagation and synaptic transmission. Together, these data demonstrate a critical window during which AP-2 is needed in the process of establishing axogenesis including axonal branching and establishing the repertoire of ion channels critical for signal propagation to synapses.

## Results

### Early depletion of AP-2 impairs proper axon extension in hippocampal neurons

**Figure 1:**
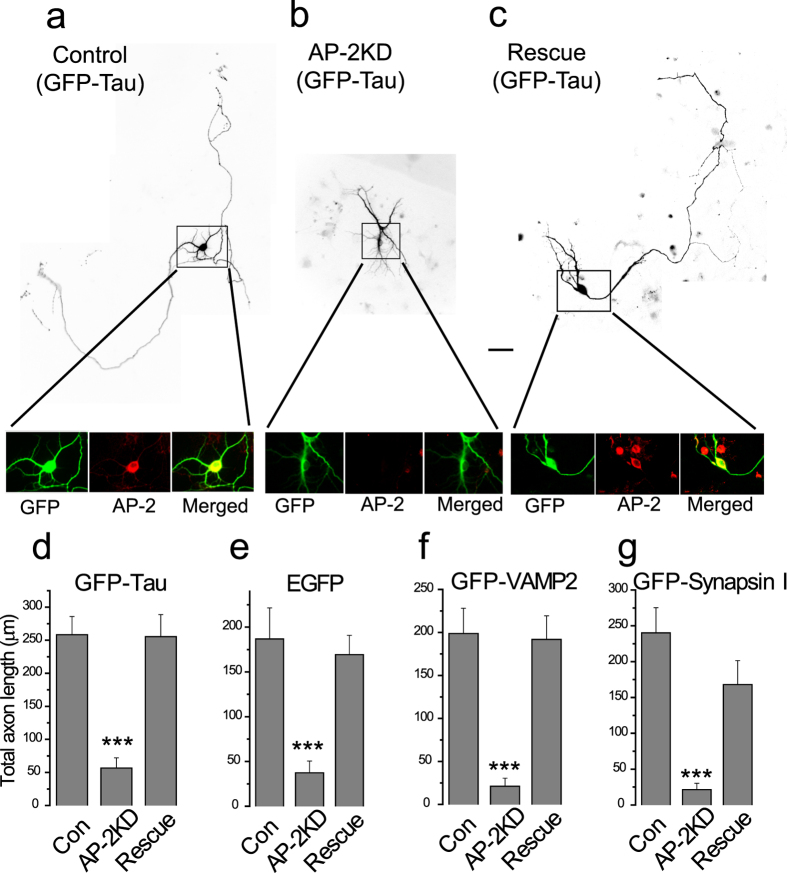
Early AP-2 depletion has defect in axon formation in hippocampal neurons. (**a–c**) Representative images of axon extension in hippocampal neurons (cultured for 7 days) expressing GFP-Tau taken from: (**a**) Control cell (expressing GFP-Tau alone), (**b**) AP-2KD cell (expressing GFP-Tau + shRNA targeting μ2 subunit of AP-2), and (**c**) Rescue cell (Expressing GFP-Tau, shRNA targeting μ2 subunit of AP-2, and shRNA-μ2 resistant μ2 plasmid). Knockdown shRNA was added at 36–48 hours after plating. (**a**–**c**) (Inset) magnified cell body of neurons that were immunolabeled with left to right: anti-GFP (green) and anti-α-adaptin AP-2 subunit antibody (α subunit of AP-2 complex), (red) and merged (overlay of red and green imaged). (**d**) Total axon length of neurons from all three conditions (Control, AP-2KD, and Rescue). Total length of axon including primary and all sub-branched axons were measured: Total length of axon; GFP-Tau _con_ = 258.3 ± 27.5 μm (n = 12), GFP-Tau _AP-2KD_ = 56.8 ± 15.2 μm (n = 18), GFP-Tau _Rescue_ = 255.39 ± 33.4 μm (n = 12), (**e–g**) Total –axon length of neurons from all three conditions (Control, AP-2KD, and Rescue) using exogenously expressed cytosolic marker (**e**; EGFP only) or axonal markers (**f**; GFP-VAMP2, (**g**) GFP-Synapsin I respectively) with the same method as (**d**). (**e**) EGFP _con_ = 186.9 ± 34.5 μm (n = 12), EGFP_AP-2KD_ = 37.4 ± 13.0 μm (n = 14), EGFP _Rescue_ = 169.3 ± 21.6 μm (n = 8). (**f**) GFP-VAMP2 _con_ = 198.8 ± 29.3 μm (n = 16), GFP-VAMP2 _AP-2KD_ = 21.1 ± 9.5 μm (n = 9), GFP-VAMP2_Rescue_ = 191.9 ± 27.4 μm (n = 10). (**g**) GFP-Synapsin I _con_ = 240.1 ± 35.2 μm (n = 10), GFP-Synapsin I _AP-2KD_ = 21.4 ± 9.0 μm (n = 13), GFP-Synapsin I _Rescue_ = 168.1 ± 33.4 μm (n = 5). Scale bar represents 10 μm. ***p < 0.001. One-way ANOVA.

Dissociated hippocampal neurons are known to go through a series of morphological changes when plated *in vitro*^26^. This includes the formation of lamellipodia, extension and retraction of short neurites and the emergence of a single rapidly growing axon. To determine if AP-2 complex used in synaptic endocytosis plays an important role in axogenesis we delivered shRNA targeting the μ2 subunit of the AP-2 protein complex to neurons ~36 hours after plating. AP-2 consists of an obligate hetero-tetramer consisting of α, β2, μ2 and σ2. Removal of a single one of these subunits in mammalian cells results in loss of the AP-2 complex^27^. The efficiency of the knockdown in individual neurons was determined by retrospective immunocytochemistry against the AP-2 α subunit as a proxy for the AP-2 complex as a whole, which showed that shRNA treatment targeting the μ2 subunit resulted in >90% loss of the AP-2 complex (Fig. 1a–c inset and supplementary Fig. 1). In order to examine the morphology of the transfected cell, we co-transfected neurons receiving shRNA with either a cytosolic marker (soluble GFP) or GFP-tagged proteins that eventually become polarized to the axon (GFP-Tau, GFP-VAMP2, GFP-synapsin I). Five days after transfection, neurons were fixed and imaged. Under control conditions (cells receiving GFP alone or a GFP-tagged protein) we observed the expected morphology for this age *in vitro*: a cell body with a number of shorter thick neuritic extensions as well as a single, often branched narrow process that extends over a large distance (Fig. 1a also see supplementary Fig. 2), which we take to be the nascent axon. However, in cells in which AP-2 had been ablated the cell body had less developed neuritic extensions (Fig. 1b) with the longest neurite on average only 25% of the axonal length in control neurons (Fig. 1a). These results were invariant with respect to the type of marker used to examine the morphology (Fig. 1d–g, see also supplementary Fig. 2). Although the axon extension was impaired, there was still a strong development of polarity for axon specific markers only in one neurite extension including, synapsin, VAMP2 and Tau proteins (supplementary Fig. 2). Proper polarization was also confirmed by sorting of endogenous microtubule associated protein 2 (MAP2), which was properly confined to the dendrites of both control and AP-2KD neurons (supplementary Fig. 2). We confirmed the specificity of the shRNA by demonstrating that these effects were fully reversed when a plasmid encoding an shRNA-resistant μ2 was co-transfected with the shRNA and the morphological marker (Fig. 1c,d). Equivalent results were obtained with all 4 of the different markers used in rescue-transfected neurons, which were indistinguishable from WT (Fig. 1d–g). Thus the defects in axonal extension resulted from AP-2 ablation through μ2 subunit deletion during early development.

**Figure 2:**
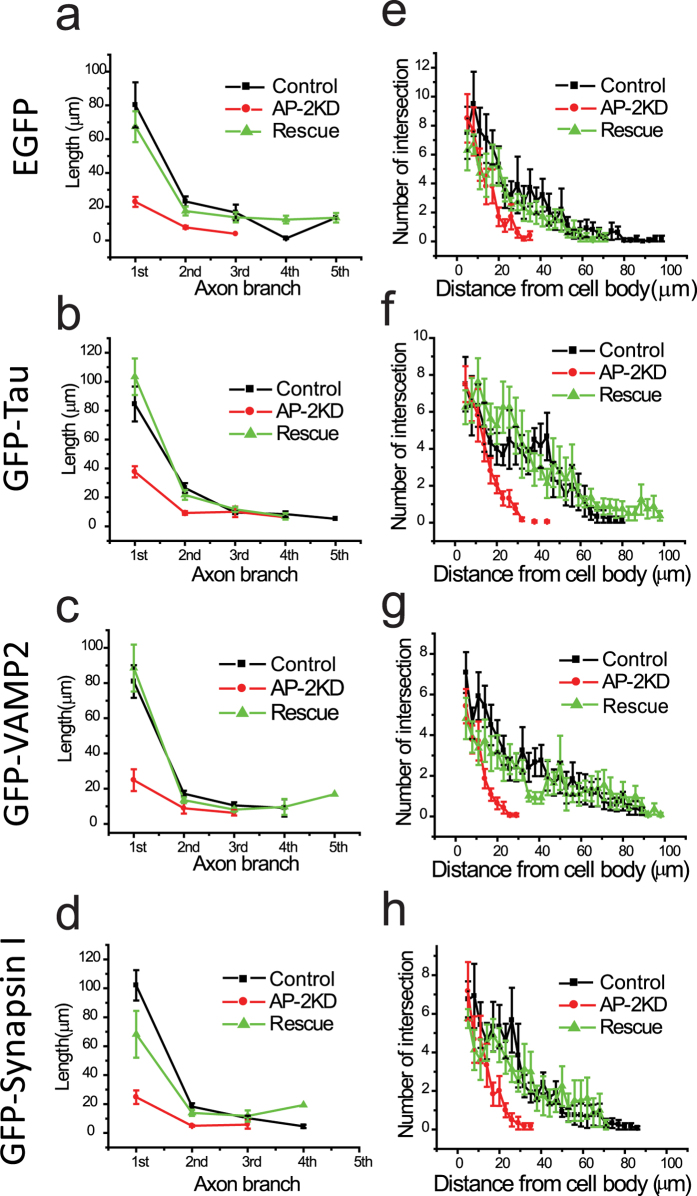
Impairment of AP-2 expression leads to a reduction in neuronal process. (**a–d**) The lengths of individual branch of axon were measured using four markers (EGFP. GFP-Tau, GFP-VAMP2 and GFP-Synapsin I). AP-2 depleted neurons have significantly deficient axon extension in each axon branch. (**e–h**) Sholl analysis was applied in WT, AP-2KD, and rescued neurons transfected four different markers. AP-2KD neuron has significantly decreased neuronal process (<40 μm) as measured by all four different markers compare to that of WT or rescued neurons (~100 μm).

In order to examine the morphological variations in a more systematic manner, we measured branching frequency and branch length using the aforementioned markers (Fig. 2a–d) and carried out a Sholl analysis (Fig. 2e–h) in the AP-2KD, WT and rescue neurons (AP-2 KD + shRNA-resistant μ2 plasmid) (Fig. 2). These data all showed that in the absence of AP-2, axons become severely blunted and form fewer branches using multiple axonal markers. We also attempted to examine neurons that had been depleted of AP-2 at early stages but kept in culture for longer periods (>14 days *in vitro*) but were unable to find transfected neurons as assayed by expression of our GFP reporters. Presumably, those neurons were not able to sustain viability, reminiscent of the of AP-2 knockout mouse phenotype^28^.

### Early AP-2 ablated neurons still undergo synaptogenesis

**Figure 3:**
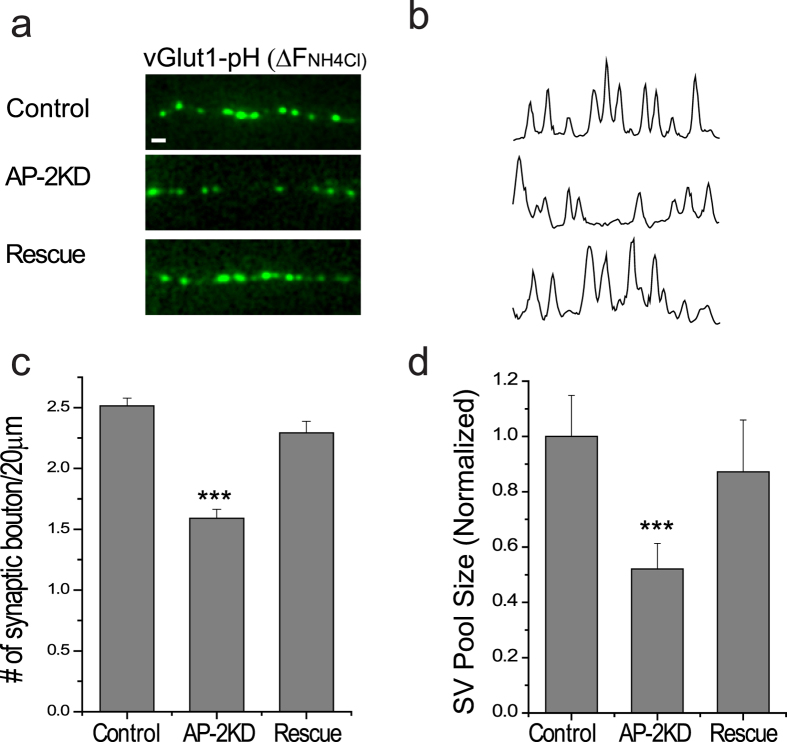
Synaptogenesis is maintained in early AP-2 depleted neurons. (**a**) Representative images of axons expressing vGlut1-pHluorin after exposure to 50 mM NH_4_Cl (pH 7.4) to reveal intracellular pool of vesicles for control, AP-2KD and rescue conditions. Scale bar represent 5 μm. (**b**) Line scans of intensity from images in (**a**). (**c**) Mean values of the number of synaptic boutons per 20 μm of axon in Control, AP-2KD, and Rescue neurons. Control = 2.51 ± 0.06/20 μm (n = 6 cells), AP-2KD = 1.58 ± 0.07/20 μm (n = 5 cells), Rescue = 2.29216 ± 0.0956/20 μm (n = 6 cells). (**d**) Quantification of the total synaptic vesicle pool as measured by NH_4_Cl application in Control, AP-2KD, and Rescue neurons expressing vGlut1-pHlourin normalized to control cells. Control = 100.0 ± 14.8% (n = 8 cells), AP-2KD = 52.1 ± 9.2% (n = 5 cells), Rescue = 87.2 ± 1.8% (n = 5 cells). ***p < 0.001. One-way ANOVA.

Following axon elongation and polarization, synaptogenesis of *en passant* boutons occurs in hippocampal neurons during development. Although we quantitatively observed less axogenesis in AP-2KD neurons, synaptogenesis still persisted. We identified synapses using the expression of a vesicle specific glutamate transporter (vGlut1) with an attached lumenal pH-sensitive GFP molecule (pHluorin). Intracellular vesicles can be visualized by a brief application of NH_4_Cl (pH7.4) that neutralized the alkaline lumen of the vesicle^29^ (Fig. 3a,b). The density of synaptic boutons (vG-pH positive puncta) measured at 7–8 days *in vitro* (DIV) was decreased by ~30% in AP-2KD axons compared to control (2.51 ± 0.06/20 μm and 1.58 ± 0.07/20 μm for control and AP-2KD neurons respectively) (Fig. 3c). In addition to having fewer boutons, the size of the synaptic vesicle pool (defined by the average maximal NH_4_Cl vG-pH response) at each bouton in AP-2KD neurons was also decreased by 50% compared to control synapses (Fig. 3d). In each case, we confirmed that these defects from early AP-2 depletion were fully restored by expressing shRNA-insensitive μ2. These results indicate that the loss of AP-2 does not preclude synaptogenesis in the truncated axon, although the density and size of boutons are decreased (Fig. 3a–d).

### Activity-driven Ca^2+^ influx and synaptic vesicle release at nerve terminal is disrupted in early AP-2 depleted neurons

**Figure 4:**
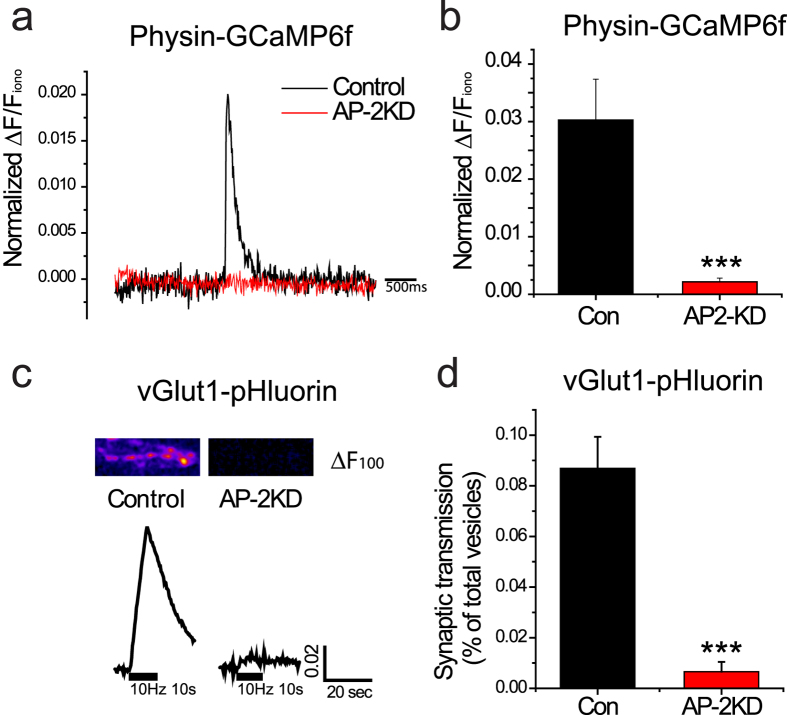
Activity-driven Ca^2+^ influx and vesicle fusion are impaired in early AP-2 depleted synapses. (**a**) Representative single action potential stimulated GCaMP6f traces in control (black) and early AP-2KD neurons (red). (**b**) Mean value of GCaMP6f 1AP response amplitudes in control and AP-2KD neuron. Con = 0.0302 ± 0.71 (n = 7 cells), AP-2KD = 0.0021 ± 0.06 (n = 8 cells). (**c**) Representative vG-pH trace of neuron in control and early AP-2KD neurons. Neurons expressing vG-pH only, vG-pH with shRNA against AP-2 were stimulated with 100 action potential at 10 Hz. (Inset) ΔF image of synaptic bouton after 100 action potential at 10 Hz. (**d**) Mean values of the amount of synaptic transmission by 100 AP. Con = 8.69 ± 1.24% (n = 8 cells), AP-2KD = 0.65 ± 0.38% (n = 7 cells). ***p < 0.001.

When an action potential invades a presynaptic terminal it activates voltage-gated Ca^2+^ (Ca_V_) channels, which catalyzes vesicle fusion and release of neurotransmitter. Next, we undertook measurements of synaptic function at the nerve terminals of AP-2KD neurons. We made use of a sensitive genetically-encoded Ca^2+^ indicator, GCaMP6f 30, fused to synaptophysin to specifically measure Ca^2+^ influx at presynaptic nerve terminals. We compared activity-driven responses to the value obtained following perfusion of ionomycin, which should saturate the probe^31^. As shown in Fig. 4a,b, activity-driven Ca^2+^ influx is absent from early AP-2KD neurons while control neurons show a robust increase even from stimulation with a single AP. Synaptic transmission is tightly regulated by Ca^2+^ influx at the nerve terminal^32^. As such, we also utilized vGlut1-pHlourin (vG-pH) system, to measure synaptic vesicle release during trains of stimulation as a sensitive assay^29^ for activation of Ca_V_ channels at nerve terminals^31^,^33^. We delivered 100 stimuli at 10 Hz. Under these conditions, control neurons released 8.69 ± 1.2% of their synaptic vesicles, whereas AP-2KD neurons showed a response just above baseline even under prolonged stimulation (0.65 ± 0.3%) (Fig. 4c,d).

### AP-2 in not essential at later stages of synaptogenesis

**Figure 5:**
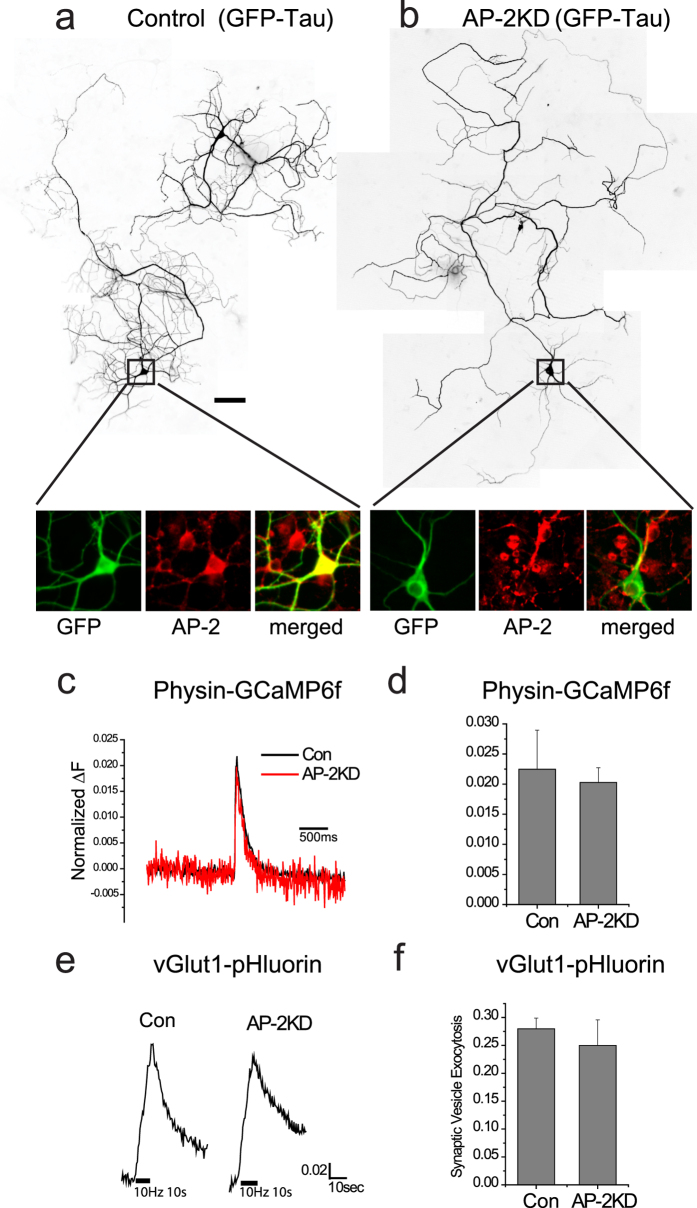
Late-stage AP-2 depletion does not impair synaptic function. (**a,b**) Representative images of axon extension in hippocampal neurons (cultured for 14–16 days) expressing GFP-Tau with as an axonal marker taken from: (**a**) Control cell (expressing GFP-Tau alone), (**b**) Late AP-2KD cell (expressing GFP-Tau + shRNA targeting μ2 subunit of AP-2). Knockdown shRNA was added at 8 DIV after plating. A-B (Inset), magnified cell body of neurons that were immunolabeled with left to right: anti-GFP (green) and anti-α-adaptin antibody (red) and merged (overlay of red and green imaged). (**c**) Representative 1 action potential stimulated GCaMP6f traces in control (black) and late AP-2KD neurons (red). (**d**) Mean value of Physin-GCaMP6f 1 action potential response amplitudes in control and AP-2KD neuron. Con = 2.24 ± 0.64% (n = 4 cells), AP-2KD = 2.02 ± 0.24% (n = 4 cells). (**e**) Representative vG-pH traces of control and late AP-2KD neurons 100 action potential pulses at 10 Hz. (**f**) Mean values of peak vG-pH in control and AP-2KD cells normalized to NH_4_Cl response. Con = 27.98 ± 0.01% (n = 11 cells), AP-2KD = 24.98 ± 4.58% (n = 8 cells), p = 0.51.

The retardation of axogenesis by early AP-2KD suggests that AP-2 is critical during the initiation period of polarization for establishing functional synaptic terminals capable of neurotransmission. In order to determine whether the dependence on AP-2 integrity is maintained at later stages of axon development we examined the consequences of AP-2KD applied to cells at DIV 7–8 and examined at >DIV 14. We previously showed that KD at this stage results in a >96% KD of AP-2^24^. Between DIV 7–8 and DIV14 the axon continues significant growth, including additional branching and elaboration preventing a full quantitative analysis as applied to the earlier developmental stage. However, neurons transfected with shRNA targeting μ2 and GFP-Tau (Fig. 5a,b) or GFP-VAMP2 (data not shown) at 7–8 DIV showed no obvious impairment in axon growth. Similar to early AP-2KD there was no impairment of polarization of tau protein in later stage AP-2KD (Fig. 5b). Analysis of Ca^2+^ influx and vesicle exocytosis did not show any impairment in late AP-2KD neurons (Fig. 5c–f), though there was an impairment in synaptic vesicle endocytosis as previously reported^24^.

### Early AP-2 depletion impairs action potential generation

**Figure 6:**
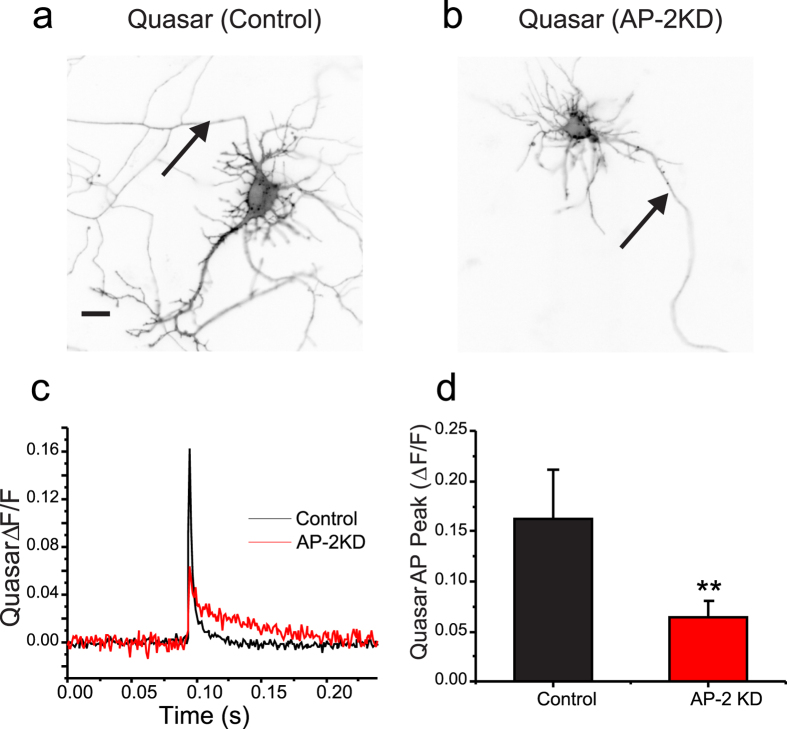
The waveform of action potentials is disrupted in AP-2 depleted neurons. (**a,b**) Representative images of Quasar transfected neurons ± AP-2KD with arrows indicating AIS. Scale bar = 5 μm. (**c**) Average traces of optically recorded action potentials from control (Black; n = 11) and AP-2KD (red; n = 13) neurons after subtraction of field stimulus, isolated by inclusion of TTX (300 nM) extracellularly, see methods. (**d**) Amplitude of AP waveforms recorded from soma of control (black; 0.177 ± 0.044) and AP-2KD (red; 0.0596 ± 0.016). **p < 0.01.

The failure of field-stimulation to elicit vesicle exocytosis or Ca^2+^ -influx at presynaptic terminals in early AP-2KD neurons raised the question of functional electrical activity and signal propagation to synaptic terminal. Recently using a genetically-encoded voltage indicator in combination with high speed imaging, it has been possible to make subcellular measurements of electrical activity in hippocampal neurons^34^. In the present study, we took advantage of the latest version of archaerhodopsin-based voltage indicator QuasAr2^35^ to examine the action potential waveform at the somas of control and early AP-2KD neurons (Fig. 6a,b). As shown in Figs 6c,d, the peak amplitude of the action potential waveform in AP-2KD neurons was severely decreased (0.05 ± 0.02 ΔF/F, n = 13) compared to control neurons (0.17 ± 0.04 ΔF/F, n = 11). Indeed, the waveform itself did not resemble an action potential with its low amplitude and lack of rapid polarization (Fig. 6c,d). Measurements at the presynaptic terminals in AP-2KD neurons did not yield any measurable signal making comparison to control cells impossible (supplementary Fig. 3). Thus, although AP-2 KD neurons establish polarity and synaptic terminals, they are unable to generate or propagate action potentials. This was not the case in mature neurons, when depleting AP-2 complex levels at later stages of development. In these cells, action potential generation and propagation was still quite robust within the distal axon (>200 μm from soma; supplementary Fig. 4) as expected from prior measurements of synaptic function when AP-KD occurs at DIV 7–8 neurons.

### Early AP-2 depletion impairs the maturation of the Axon Initial Segment (AIS) in hippocampal neuron

**Figure 7:**
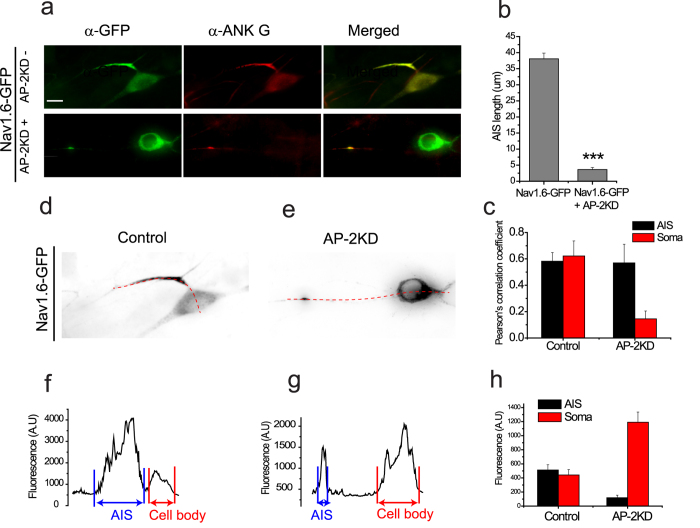
AP-2 ablation impairs the formation of axon initial segment. (**a**) Representative images of cells transfected at 2 DIV with Na_v_1.6-GFP ± shRNA against AP-2 (AP-2KD). Neurons were fixed at 7 DIV and incubated with anti-GFP (green) and anti-ankyrin G (red). Arrow indicates AIS. (Inset) magnified images of AIS region to clearly visualize. (**b**) Mean value of the length of the AIS in each condition. Na_v_1.6-GFP only = 38.09 ± 1.75 μm (n = 24 cells), Na_v_1.6-GFP + AP-2KD = 3.61 ± 0.65 μm (n = 17 cells). ***p < 0.001. (**c**) Mean value of Pearson’s co-localization coefficient of Na_v_1.6-GFP and AnkyrinG in AIS and Soma. Control _AIS_ = 0.58 ± 0.07, Control _soma_ = 0.62 ± 0.11. AP-2KD_AIS_ = 0.57 ± 0.14, AP-2KD_soma_ = 0.15 ± 0.59. (**d–h**) Measurement of Na_v_1.6-GFP at AIS and soma in control and AP-2KD neuron. (**d,e**) Dashed lines indicate line scan of Na_v_1.6-GFP distribution from AIS to Soma. (**f** and **g**) Profiles of line scan of Na_v_1.6-GFP in control (**f**) and AP-2KD (**g**) neurons. (**h**) Mean values of density of Na_v_1.6-GFP at AIS and soma in control and AP-2KD neurons. Control _AIS_ = 513.63 ± 72.69 a.u., Control _soma_ = 442.51 ± 76.19 a.u. (n = 21 cells). AP-2KD_AIS_ = 119.43 ± 35.23 a.u., AP-2KD_soma_ = 1190.86 ± 145.63 a.u. (n = 15 cells).

In addition to axonal elongation and synaptogenesis, another important aspect of axogenesis is the generation of specialized signaling domains with voltage-gated ion channels. One such domain is the AIS, a structural and functional hallmark of the axon that both maintains polarity and it the site for action potential initiation. Structurally the AIS is composed of several ion channels (e.g. Na_V_ channels) and structural proteins such as ankyrin G (AnkG)^36^. It has previously been demonstrated that the silencing AnkG dismantles the AIS and causes axons to acquire the molecular characteristics of dendrites^37^. Although we didn’t observe changes in polarity with early AP-2KD, we reasoned that the maturation of the AIS where other components with AnkG binding motifs including sodium channels interact with AnkG might be impaired^38^. In dissociated hippocampal neurons the AIS appears four days after plating *in vitro* (supplementary Fig. 5)^39^. We monitored AIS formation for control and AP-2KD neurons using AIS markers. Given the importance of Na_V_ channels for signaling in AIS, we measured their trafficking by expressing a functional exogenous Na_V_1.6 channel tagged with GFP^40^. In control neurons expressing Na_V_1.6-GFP only, we measured a distinct segment of sodium channels colocalized with AnkG measuring 38.09 ± 1.75 μm (Fig. 7a,b) in close agreement with previous reports^41^. However, in AP-2KD neurons it was hard to detect any significant enrichment in AP-2 depleted neurons with enrichment less than 10% of control neurons, Na_V_1.6-GFP + AP-2KD = 3.61 ± 0.65 μm (Fig. 7a,b). Intriguingly, Na_v_1.6 is easily visible in the soma of AP-2KD suggesting a defect in trafficking of the channel not its stability. As shown in Fig. 7c–h, Na_v_1.6-GFP is enriched at the cell body of AP-2KD neurons (2-fold higher than control), whereas Na_v_1.6-GFP is distributed more at the AIS than at cell body of control neurons (Fig. 7c). Although knockdown of AP-2 doesn’t alter polarization in a similar manner to AnkG silencing (supplementary Fig. 6), there is a total impairment of action potential initiation. These results suggest that AIS maturation and Na_v_1.6 trafficking to the AIS are mediated by AP-2.

## Discussion

**Figure 8:**
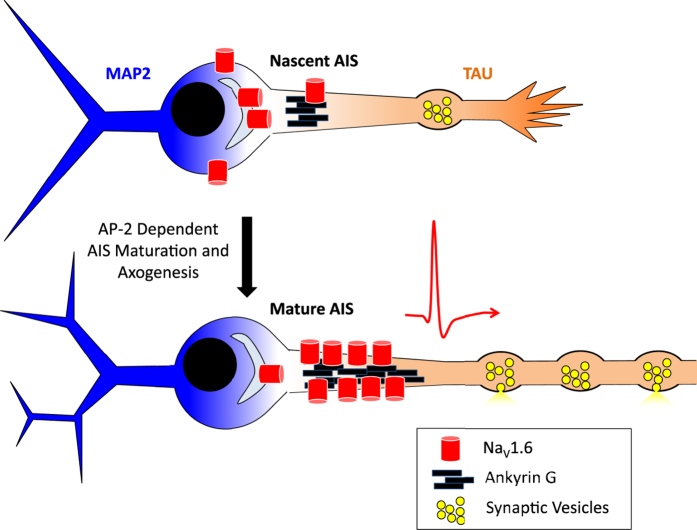
Model of axogenesis in an early AP-2 depleted neuron. Suggested model of axogenesis with and without AP-2 present. AP-2 mediated endocytosis is essential for establishing and maintaining axon elongation. Furthermore, AP-2 plays a critical role for establishing a functional AIS, the loss of which leads to impaired synaptogenesis and signal transduction.

In the present study, we demonstrate that AP-2 has a unique role in the early development of the neuron that is critical for establishing a functional axon. We characterize three distinct phenotypes of neurons that lack AP-2 at the early stage of development: First, impaired extension and arborization of the axon; Second, failure of the axon initial segment to elongate and mature with functional enrichment of Na_V_ channels; and third, the defects in action potential generation and propagation (Fig. 8). The well-studied development of the AIS has always displayed a tightly aligned temporal sequence of protein enrichment anchored by AnkG that has always maintained two important but unique roles that have previously seemed intertwined, maintaining polarity and transforming synaptic inputs into action potentials. Interestingly, it seems that AP-2 is necessary for maturation of the nascent AIS and accumulation of proteins important for electrical signaling in young neurons. This role for AP-2 in the development of the AIS is independent of establishing polarity as axonal proteins still exclusively clustered into axons (Fig. 1 and supplementary Fig. 2) even with a truncated AIS (Fig. 7). Here, we show a unique role for AP-2 in establishing electrical signaling and clustering Na_V_ channels at the axon initial segment that is independent of polarization at the AIS. To our knowledge this is the first time that these two roles have been independently observed in development.

The adaptor complex AP-2 is the major clathrin-associated adaptor for cargo recognition at the plasma membrane. There is accumulating evidence that endocytosis contributes to the formation of cellular polarity by proper sorting and localizing key molecules in a number of tissue types^20^,^21^,^22^. In our results, neurons display a deficit in axon extension when AP-2 complex levels are depleted at the early period of cellular development, but these axons still go on to form synaptic boutons. Indeed, tau, VAMP2 and synapsin still segregate from the other neurites of the young neuron (Fig. 1). This likely indicates that the role of the AIS for modulating trafficking of synaptic components is maintained in its depleted state, but not its role for integrating synaptic activity to fire action potentials. The observed loss of physiological responses at presynaptic terminal to electrical stimulation are dramatically impaired without any appreciable Ca^2+^ influx or vesicle fusion when stimulated in AP-2KD neurons. We attribute these impairments to the lack of propagating action potentials that are likely caused by the truncated AIS as measured by AnkG and a failure to establish significant concentrations of Na_V_1.6 at the AIS (Fig. 7).

The AIS is composed of a host of proteins that subsequently enrich at the AIS including cytoskeletal proteins, cell adhesion molecules (CAMs) and ion channels. The first two proteins to localize to the AIS are βIV-spectrin and AnkG. The establishment of AnkG is followed by the localization of Na_V_ 1.6 channels and the L1-CAMS (neurofascin and NrCAM) as the AIS matures with varying dynamics^38^,^42^. These varied modes of enrichment and maturation suggest different modes of trafficking of AIS components. Although it has been well established that AnkG is necessary for clustering sodium channels^36^,^40^, it has also been demonstrated that knocking down Na_V_ channels in early stages of neural development destabilized the AIS in motor neurons^43^. Since the loss of AP-2 proteins mimics the loss of Na_V_ channels we think that AP-2 may contribute to an endocytosis pathway that plays a critical role in the formation of the AIS. Furthermore, the loss of Na_V_ channel enrichment halts the subsequent addition of other proteins that make up the functional AIS.

Due to technical limitations, we were not able to trace Na_V_ or AnkG protein translocation and detect a role for AP-2 mediated endocytosis and targeted delivery. Recent work suggests that Na_V_ channels are delivered by exocytosis to the AIS^44^. There are a number of receptors and other proteins whose endocytosis is required for axogenesis. One recently described pathway that initiates axogenesis involves the retrieval of activated type II TGFβ receptor^1^. This receptor is known to directly associate with AP-2 through the β2 subunit^45^. One possible explanation of the acute requirement for endocytic function at early developmental stages is that successful signaling to initiate and maintain a preliminary axogenesis program requires internalization of the TGFβ ligand-receptor complex, perhaps to generate a signaling endosome. Another example of AP-2 modulated signaling during development involves the establishment of planar cell polarity^46^,^47^, and neurite outgrowth by Wnt/Frizzled^2^. AP-2 with Dishevelled, a downstream hub molecule of Wnt/Frizzled signaling, appears to regulate endocytosis of the Frizzled receptor and contribute to planer cell polarity^47^,^48^. Our results indicate that AP-2 is also involved in separate sorting for establishing an essential component of neuronal polarity and excitability, the AIS. It is well known that a number of voltage-gated ion channels are properly sorted by interacting with AP-2 complex^20^,^49^. It is likely that early depletion of AP-2 disrupts the correct sorting or targeting of voltage-gated ion channels to the AIS, and eventually destabilizes the other structural and transynaptic proteins that accumulate at the AIS. Specific antibodies that could recognize endogenous Na_V_1.6 channels with high specificity were not available for immunostaining. We used a low expression plasmid to express a functional Na_V_1.6-eGFP as a proxy for endogenous channel trafficking^40^. Although our result using this exogenous proteins shows that Na_V_1.6 clearly fails to accumulate to the AIS, it is still unclear if Na_v_1.6 itself is directly retrieved by AP-2, and if this may also be the case for other voltage-gated ion channels. The fact that Na_V_1.6 accumulates in the somata of AP-2KD neurons does indicate that retrieval and delivery rather than simple diffusion likely plays a critical role for establishing a concentration of channels at the AIS (Fig. 7c–h). We cannot exclude the possibility that Ca_V_ channel targeting to presynaptic terminals as well as other channels are also modulated by AP-2. However, the propagation of action potentials is upstream of Ca^2+^ entry and K^+^ channel activation masking other deficits at terminals.

Although cargo-internalization during endocytosis is a principal function of AP-2, we cannot rule out that other mechanisms for this protein or the μ2-subunit at the AIS. Indeed, studies in C. elegans have demonstrated a role for hemicomplexes of AP-2^50^. Additionally, the mechanisms for AnkG delivery to the AIS is unknown, as such our results also cannot rule out an important role for AP-2 to maintain or enrich AnkG at the AIS, which could also account for the observed deficiencies in the accumulation of Na_V_ channels. AnkG plays an important role as an adaptor for motor protein entrance and delivery of cargo to the AIS, so a specific disruption to this process by AP-2 KD could account for the observed deficits in AIS maturation and axogenesis^51^.

The lack of phenotype in later stages of development with AP-2KD suggests the existence of alternate endocytic or other trafficking pathways that only arise in later developmental stages. Alternatively the lack of phenotype suggests that endocytosis is only needed a low rate for maintenance that we are insensitive to in our experiments as we only observe a short 2-week window that may not fit with the turnover of these proteins. We attempted to explore this is late stage neurons DIV18-21, but still found action potential generation and propagation to be present (supplementary Fig. 4). There were small changes in the shape of the action potential during repolarization, but initiation at the AIS was clearly unperturbed.

Despite the fact that all of our results are from an *in vitro* system we believe that it provides a good conceptual framework for axogenesis and formation of the AIS. Because *in vitro* cultured hippocampal neurons have originally already formed neuronal polarity before dissected, it might still retain information for axogenesis and in a sense, axon regeneration. Our molecular manipulation demonstrates that AP-2 has a critical role in the early developmental window of axogenesis and maturation of the AIS.

## Methods

### Cell culture and immunofluorescence

Hippocampal CA3-CA1 regions were dissected from 0–1 day old Sprague Dawley rats, dissociated, and plated onto poly-ornithine-coated glass for 7–9 or 14–20 days as previously described^24^. Animal treatment was carried out in accordance with the Animal Care and Use Guidelines by Kyung Hee University. All experiments were approved by the Animal Care Committee of the Kyung Hee University. To make early stage knock down of AP-2, shRNA-μ2 with other plasmids were transfected 36–48 hours after plating. Neurons were fixed 7 days *in vitro* (DIV) after plating (5 days after transfection). To make late stage knock down of AP-2, constructs were transfected 7–8 days after plating and were fixed 14–16 DIV after plating (6–7 days after transfection). Neurons were fixed with 4% paraformaldehyde and permeabilized with 0.2% Triton X-100, blocked with 5% BSA, and subsequently incubated with appropriated primary antibodies [anti-GFP (invitrogen), anti-α-adaptin (ABR) (subunit of AP-2), anti-MAP2 (Millipore), anti-ankyrin G (Neuromab)]. Alexa 488 or Alexa 546 conjugated secondary antibodies (Invitrogen) were applied in primary antibody incubated samples with different color combinations as needed.

### Optical Setup and imaging

For immunofluorescence, images of fixed cells were acquired with using an epi-fluorescence microscope through a 20x (0.75 NA) or 40x (1.3 NA) Fluar Zeiss objective a recorded on an EMCCD camera (Andor iXon +, Model # DU-897E-BV) or PL APO 63X (1.32 NA) Leica objective (Leica DMRBE) with CoolSNAP HQ camera (Photometrics) driven by Metamorph software. Images of entire neurons were obtained by tiling images of several different fields of view with partial overlap and stitched together using Image J plugin (Stitching 2D, http://pacific.mpi-cbg.de/wiki/index.php/Stitching_2D/3D).

For action potential waveform imaging, specimens of Quasar transfected neurons were illuminated by a 637 nm laser 140 mW (Coherent OBIS Laser) with HQ620/60x and 660LP dichroic (Chroma) through a 40 × 1.3NA Zeiss Fluar Objective and a custom beam-expander to provide a ~25 μm diameter spot with a final power density of ~1850 W/cm^2^. Archaerhodopsin fluorescent emission was collected through a HQ700/75 m filter (Chroma) and captured with an IXON3 897 Ultra camera (Andor) in a cropped sensor mode (10 MHz readout, 500 ns pixel shift speed) to achieve 1 kHz frame rate imaging (exposure time of 985 μs) over an 85 × 45 pixel area with the use of an intermediate image plane mask (Optomask, Cairn Research distributed by Andor) to prevent light exposure of non-relevant pixels. Light was collected through an HQ700/75 m filter (Chroma). All voltage imaging experiments were carried out at 34 °C.

For presynaptic terminal live imaging for synaptic vesicle release and Ca^2+^ entry, constructs (vG-pH with/without shRNA-μ2 or synaptophysin-GCaMP6f with/without shRNA-μ2) were transfected 1.5~2 days or 8 days after plating. Experiments were carried out either 7–8 DIV for young neurons or 14–20DIV for mature neurons after plating. Coverslips were mounted in a stimulation chamber with laminar-flow perfusion on the stage of a custom-built laser-illuminated epi-fluorescence microscope. Live images were acquired with an Andor iXon Ultra 897 (Model # DU-897U-CS0-#BV) back-illuminated EM CCD camera. A diode pumped OBIS 488 laser (Coherent) shuttering by synchronizing TTL on/off signal from EMCCD camera during acquisition was utilized as a light source. Fluorescence excitation/emission and collection was achieved using a 40x (1.3 NA) Fluar objective lens using 500–550 nm emission and 498 nm dichroic filters (Chroma) for pHluorin and GCaMP6f. Action potentials were evoked by passing 1 ms current pulse through platinum-iridium electrodes form an isolated current stimulator (World Precision Instruments). Neurons were perfused in a saline based buffer containing (in mM) 119 NaCl, 2.5 KCl, 2 CaCl_2_, 2 MgCl_2_, 25 HEPES, 30 glucose, 10 μM 6-cyano-7nitroquinoxaline-2,3-dione (CNQX), and 50 μM D,L-2-amino-5-phosphonovaleric acid (AP5) (adjusted to pH 7.4). Temperature was clamped at 30 °C to remove effect from temperature fluctuation. All chemicals were purchased from Sigma, unless noted. NH_4_Cl solution applications were done with 50 mM NH_4_Cl in substitution of 50 mM of NaCl (pH7.4). Imaging for vG-pH, vG-pH with/without shRNA-μ2 transfected neurons were stimulated for 10 s at 10 Hz. Subsequently, NH_4_Cl was applied to measure total synaptic vesicle pool. Images were acquired at 2 Hz by exposing 50 ms. Imaging for Physin-GCaMP6f, Physin-GCaMP6f with/without shRNA-μ2 transfected neurons were stimulated with 1 AP with 2 mM external Ca^2+^. Imaging of Ca^2+^ influx by 1AP was recorded at 100 Hz by exposing 9.73 ms in frame transfer mode. Ionomycin 200 μM (Alomone Labs) was applied to record maximum fluorescence of Physin-GCaMP6f at the end of each experiment.

### Image analysis

Image J was utilized for all image analysis (http://rsb.info.nih.gov/ij). Counting and measurement of axonal length were performed using Image J plugin Neuron J.

(http://www.imagescience.org/meijering/software/neuronj), and sholl analysis was carried out using a Sholl analysis plugin (http://www-biology.ucsd.edu/labs/ghosh/software/). The longest axon directly originating from cell body was defined as the primary axon. Subsequent branches were designated in their order of appearance. Some images were displayed as inverted grey or pseudo-color to show neuronal morphology. To analyze the length of the AIS, axon containing an ankyrin G positive area was selected by line scan. The AIS was defined as the region over which the line-scanned signal was 5 times higher than standard deviation of the baseline of axon signal.

For analysis Quasar, Quasar images were also analyzed in Image J by using custom-written plugins (http://rsb.info.nih.gov/ij/plugins/time-series.html) as previously described^34^. Briefly, stimulation with 300 nM TTX present was used to isolate the field stimulation from AP signal, and was subtracted from our AP recordings at the soma and bouton. ΔF/F values of the AP were calculated after background subtraction. Measurements of background were measured using a square 10 × 10 pixel ROI. Automated peak and FWHM analyses were carried out using Origin Pro 8′s (Northampton, MA) peak fitting algorithm.

For analysis pHluorin and GCaMP6f, we followed previously described methods^31^ with minor modifications. Images were analyzed using Image J with the plugin Time-series analyzer. Fluorescence traces were analyzed with Origin Pro 8.0. For synaptic vesicle release, vG-pH positive boutons are selected as a region of interest. The peak amplitude at 100AP (ΔF values of each 100AP responses) was taken and normalized by maximum value of NH_4_Cl (total synaptic vesicle pool). For Ca^2+^ entry, Physin-GCaMP6f positive boutons were used to mark ROIs. Amplitudes of 1AP response were taken by averaging 5–6 trials. The peak values are normalized by peak of ionomycin fluorescence. Silent boutons were defined as previously described that boutons where the vG-pH _ΔF 100_ or Physin-GCaMP6f _ΔF 1AP_ was smaller than one standard deviation of the baseline before stimulation.

For statistical analysis, either one-way ANOVA (over three samples) or student t-test (two samples) was applied. Data are presented as means ± S.E.M (standard error of mean).

### Molecular biology and others

shRNA against μ2 and HA-μ2 plasmids resistant to shRNA μ2 were constructed as previously described^24^. 60-mers oligonucleotides containing rat μ2 subunit cDNA target sequences (*GTGGATGCCTTTCGCGTCA*) of AP-2 were synthesized, annealed, and ligated into pSUPER vector according to manufacturer’s instruction. HA-μ2 construct was generated using Quick Change Site-Directed mutagenesis (Stratagene). The target sequence for shRNA μ2 was mutated to *GTGGACGCTTTCCGGGTCA* without the amino acid sequence change. EGFP-C2 is from clontech, GFP-Tau, VMAP2-GFP, GFP-synapsin-1, Na_v_1.6-GFP was cloned as previously described^52^. GCaMP6f from Addgene^30^ is conjugated to synaptophysin.

## Additional Information

**How to cite this article**: Kyung, J. W. *et al*. Adaptor Protein 2 (AP-2) complex is essential for functional axogenesis in hippocampal neurons. *Sci. Rep.* **7**, 41620; doi: 10.1038/srep41620 (2017).

**Publisher's note:** Springer Nature remains neutral with regard to jurisdictional claims in published maps and institutional affiliations.

## Supplementary Material

Supplementary Information
