# Supplementary material for: Adaptor Protein 2 (AP-2) complex is essential for functional axogenesis in hippocampal neurons
Source: Sci Rep. 2017 Jan 31;7:41620. doi: 10.1038/srep41620 (PMC5282494; doi:10.1038/srep41620)
Supplement: Supplementary Information [file srep41620-s1.pdf]

Supplementary Information

Adaptor Protein 2 (AP-2) complex is essential for functional axogenesis in hippocampal neurons.

Jae Won Kyung[1], In Ha Cho[2], Sukmook Lee[3], Woo Keun Song[4], Timothy A Ryan[5], Michael B Hoppa[2]*, Sung Hyun Kim[1,6]*

[1]Department of Biomedical Science, Graduate School, Kyung Hee University, Seoul, 02447, South Korea
[2]Department of Biology, Molecular Cellular Biology Program, Dartmouth College, Hanover, NH, 03755, USA
[3]Laboratory of Molecular Cancer Therapeutics, Scripps Korea Antibody Institute, Chuncheon, 24341, South Korea
[4]School of Life Science, Bioimaging Research Center, Gwangju Institute of Science and Technology (GIST), Gwangju, 61005, South Korea
[5]Department of Biochemistry, Weill Cornell Medical College, New York, NY, 10065, USA
[6]Department of Physiology, Neurodegeneration Control Research Center, School of Medicine, Kyung Hee University, Seoul, 02447, South Korea

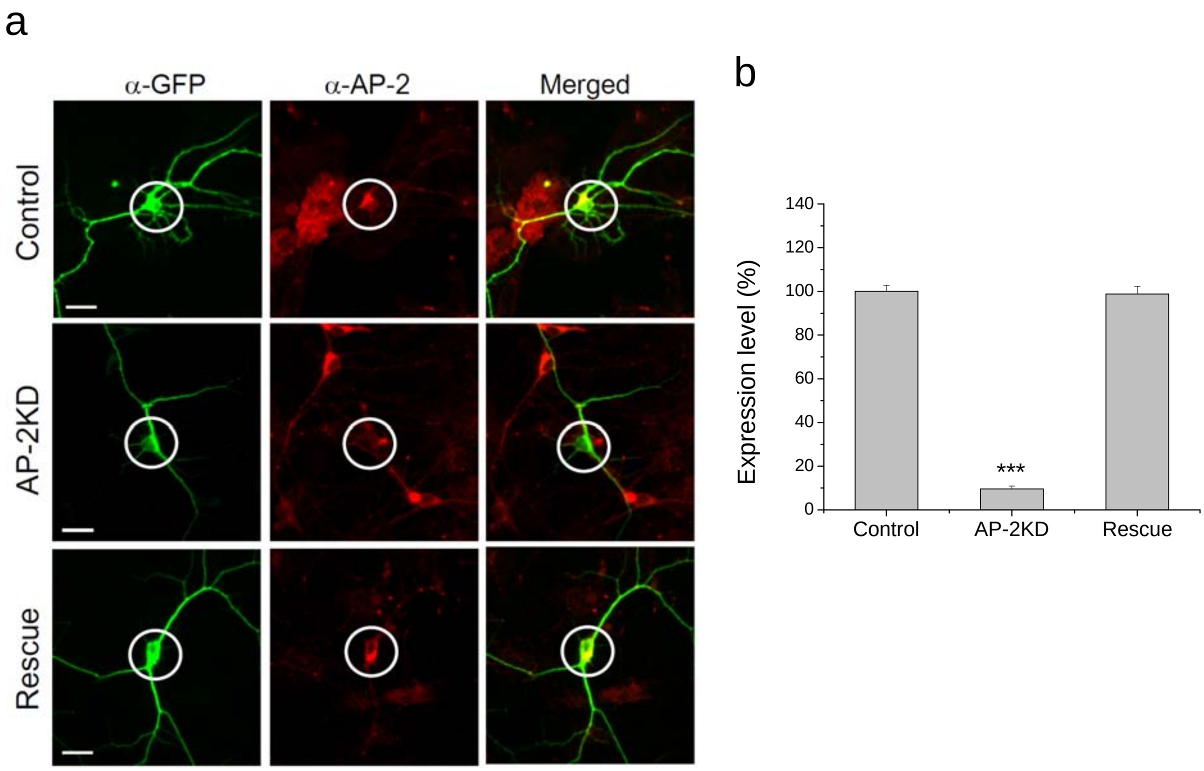

Supplementary Figure 1. AP-2 is efficiently knocked-down by introducing shRNA-μ2 in young neurons. (a) Representative images of control, AP-2KD, and rescue neurons stained with α-GFP and α-AP-2 (α-adaptin; AP-2 subunit). Transfections took place 36 hours after plating with: GFP only (control); GFP with shRNA μ2 (AP-2KD); or GFP, shRNA μ2, and shRNA resistant μ2 (rescue). At 7 days in vitro, neurons were fixed with 4% PFA and permeabilized with 0.2% Triton X-100, subsequently treated with anti-GFP and anti-α-adaptin. Alexa-488 or Alexa-546 labeled secondary antibodies were incubated. (b) Mean values of expression level of AP-2 complex in control, AP-2KD, rescue neurons at 7DIV. Cell bodies of GFP-positive neurons were selected and measured intensities of AP-2. Expression levels of AP-2 complex as assayed by immunostaining for the α-adaptin subunit decreased by introducing shRNA-mu2 and its expression is rescued by introducing an shRNA insensitive μ2 construct. Scale bar=10 μm. ***$p<0.001$.

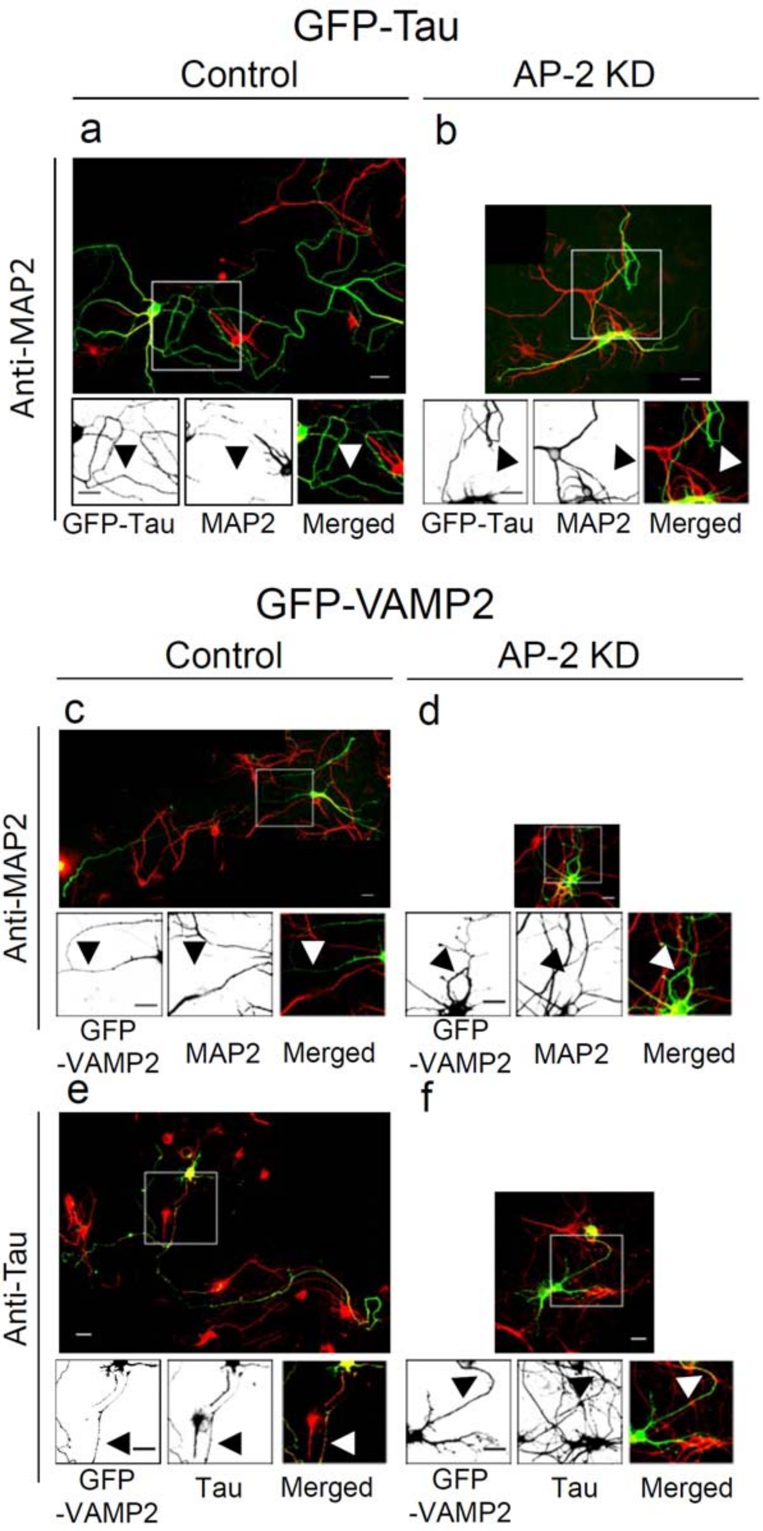
GFP-Tau
Control
AP-2 KD
a
b
Anti-MAP2
GFP-Tau
MAP2
Merged
GFP-Tau
MAP2
Merged
GFP-VAMP2
Control
AP-2 KD
c
d
Anti-MAP2
GFP
-VAMP2
MAP2
Merged
GFP
-VAMP2
MAP2
Merged
e
f
Anti-Tau
GFP
-VAMP2
Tau
Merged
GFP
-VAMP2
Tau
Merged

Continued

Continued

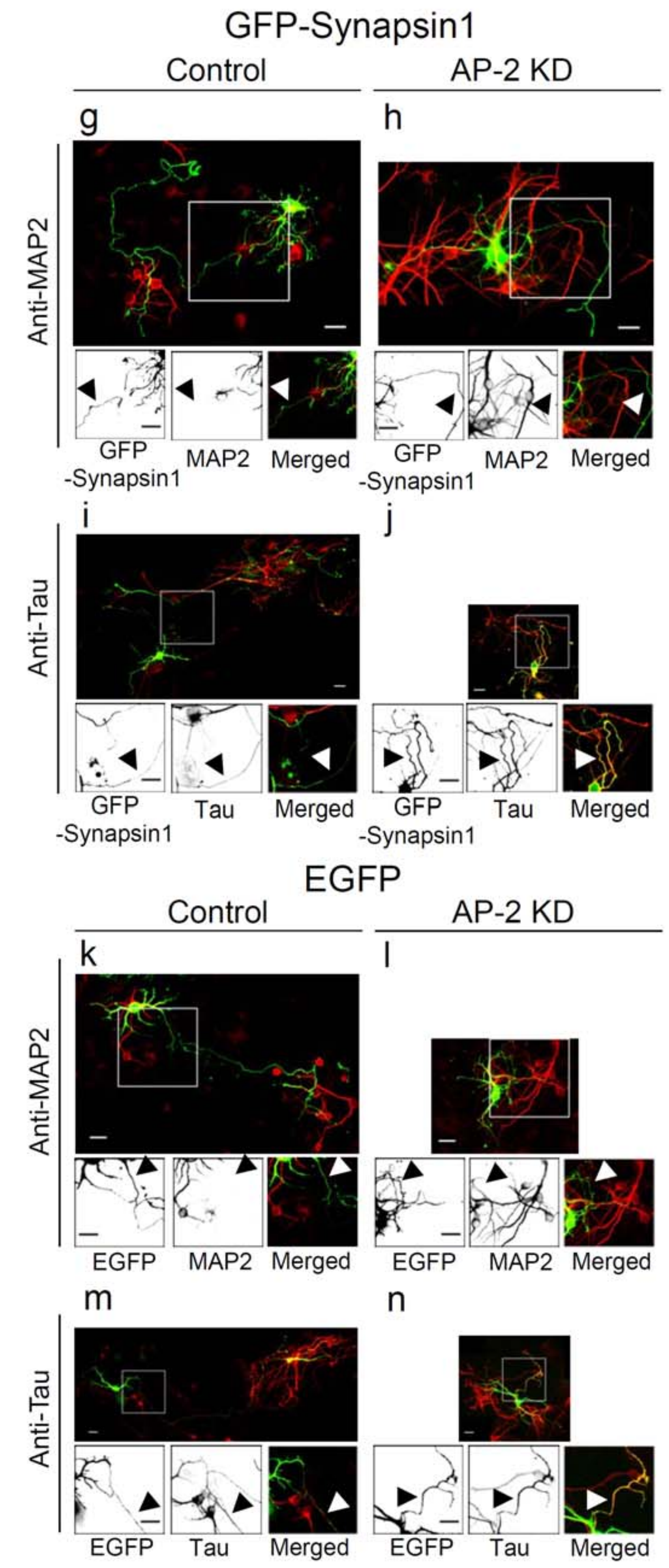
GFP-Synapsin1
Control
AP-2 KD
g
h
Anti-MAP2
GFP -Synapsin1
MAP2
Merged
i
j
Anti-Tau
GFP -Synapsin1
Tau
Merged
EGFP
Control
AP-2 KD
k
l
Anti-MAP2
EGFP
MAP2
Merged
m
n
Anti-Tau
EGFP
Tau
Merged

Supplementary Figure 2. Early depletion of AP-2 causes impairment of axon growth.

Neurons were transfected 2 days after plating with axon specific markers (a-b: GFP-Tau, c-f: GFP-VAMP2, g-j: GFP-synapsin I) or cytosolic marker (k-n: EGFP) with/without shRNA-mu2 (AP-2 subunit). Neurons were fixed at 7 days in vitro and subsequently stained with dendritic marker (anti-MAP2: a, b, c, d, g, h, k, and l, respectively) or axonal marker (anti-Tau: e, f, I, j, m, and n, respectively). (Inset: magnified box area to display clearly each channel). Arrow head indicates proper polarized axon. Early AP-2 depleted neurons impaired proper axon extension. scale bar = 10 μm

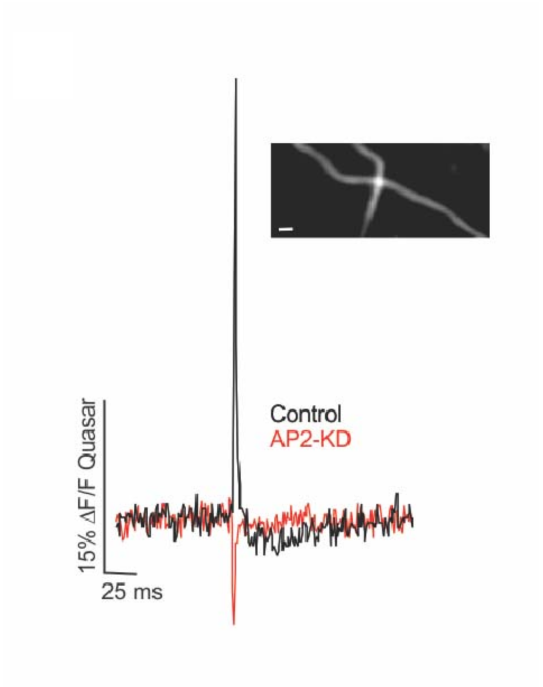

Supplementary Figure 3. Failure of action potential waveform propagation at distal axon. Representative recordings from the axon of a Quasar transfected neuron ± shRNA AP-2 (trial average 200 individual stimulations to improve the signal to noise). Note the small downward deflection of recorded membrane potential from the field stimulus pulse from the red trace demonstrating the sensitivity of Quasar, but a failure propagate any type of waveform. Inset shows the recording area of axon expressing Quasar and illuminated with 637 nm light used for the red trace in Quasar + shRNA AP-2. Scale bar = 1 μm

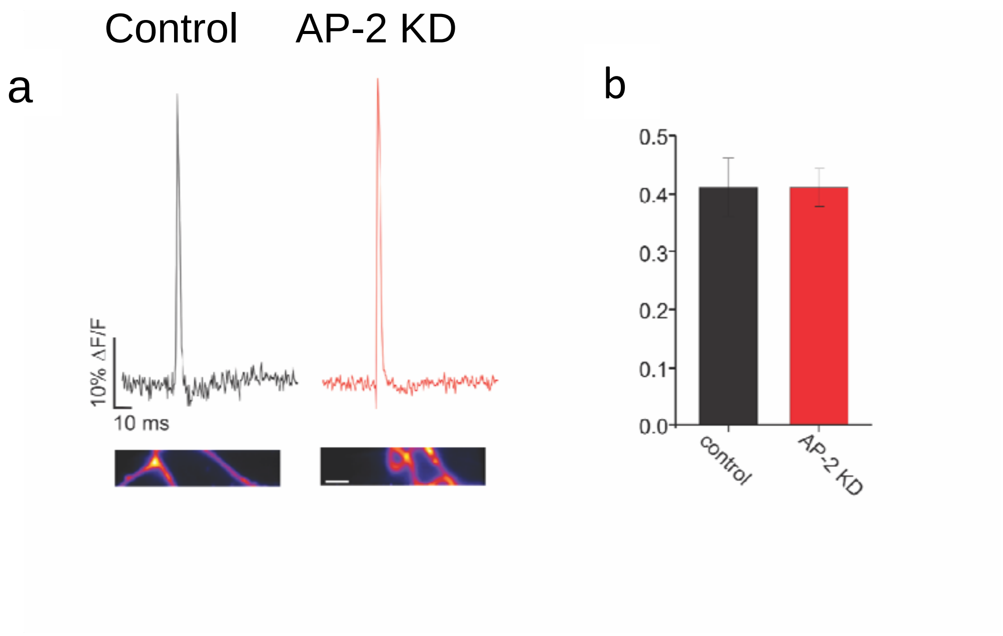

Supplementary Figure 4. Action potential waveform is not impaired in Late-stage AP-2 depleted neurons.

(a) Representative images of distal axons expressing QuasAr ± AP-2KD recorded at 2kHz from DIV 19 neurons and corresponding QuasAr signal from a single field stimulus (average response from 100 individual stimulations). (inset) Imaging windows shown below traces; Scale bar = 4µm. (b) Amplitude of AP waveforms recorded from soma of control (n=3, black; 0.41±0.050) and AP-2KD (n=5, red; 0.41±0.033).

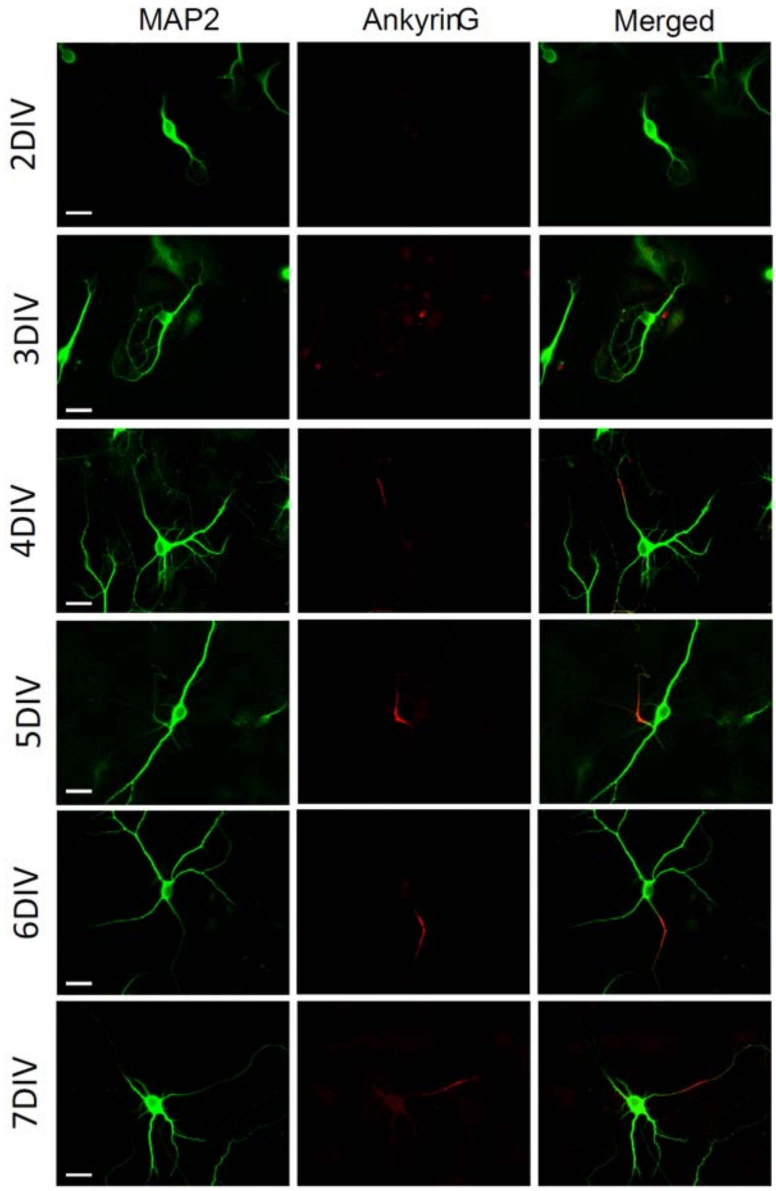

Supplementary Figure 5. The formation of axon initial segment (AIS) appears at 4 DIV in primary cultured hippocampal neurons.

Immunostained neurons grown from 2-7 days in vitro prior to fixation. Subsequently cells were stained with anti-MAP2 and anti-ankyrin G antibodies to visualize AIS on axon. AIS first appears at 4 DIV in primary cultured hippocampal neurons. Arrow indicates AIS. Scale bar = 10 μm

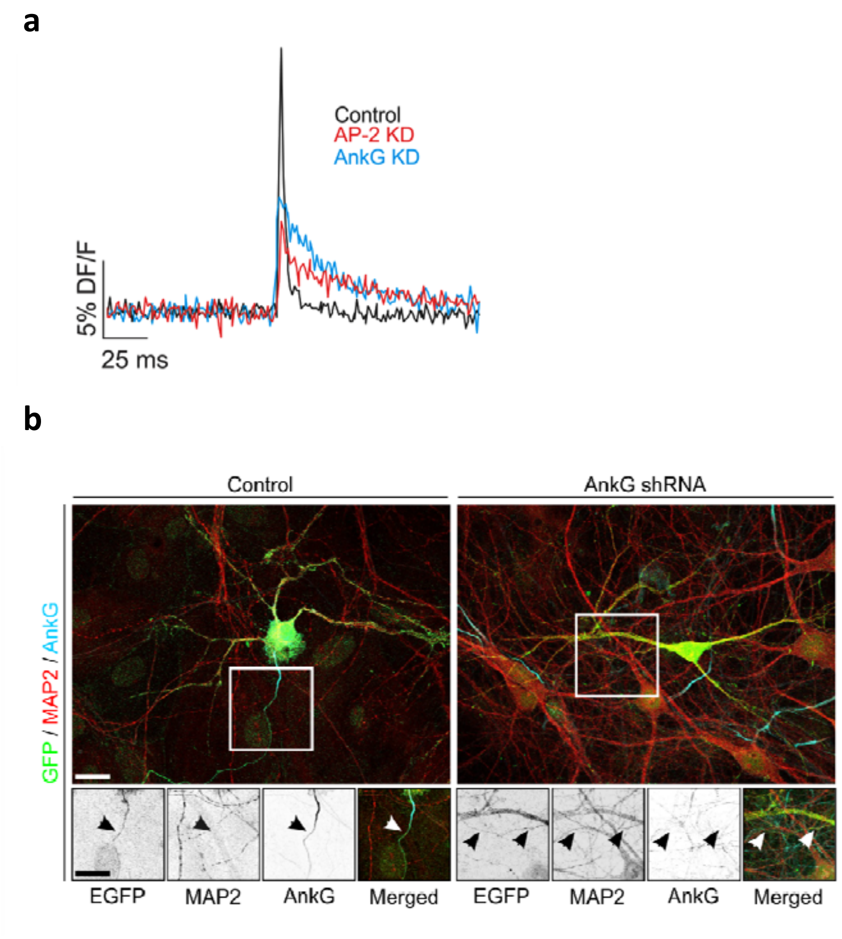

Supplementary Figure 6. Early Ankyrin G depletion causes impairment of waveform of action potential in hippocampal neurons.

(a) Average action potential waveforms recorded from the soma of neurons stimulated by 1 action potential pulse in control neurons (black, n=11), AP-2KD neurons (red, n=13) and Ankyrin G (AnkG) KD neurons (cyan, n=7). (b) Representative images from neurons expressing GFP±shRNA AnkG after fixation and immunostaining for GFP, Ankyrin G and MAP2 respectively. White box and inset is focused on the AIS, identified by black arrows. Note the small amount of MAP2 penetrating the AIS of AnkG neurons at DIV16. Scale bar = 20 μm
